# Acute whole-body vibration as a recovery strategy did not alter the content of gluteus medius monocarboxylate-transporters, lactatemia, and acidosis induced by intense exercise in horses

**DOI:** 10.3389/fvets.2025.1538195

**Published:** 2025-03-06

**Authors:** Júlia Ribeiro Garcia Carvalho, Nathali Adrielli Agassi Sales, Thayssa Oliveira Littiere, Guilherme Barbosa Costa, Catarina Mariano Castro, Emanuel Elias Camolese Polisel, Juan Bordon Orsi, Gabriel Vieira Ramos, Ivan Felismino Charas Santos, Claudio Alexandre Gobatto, Fúlvia Barros Manchado-Gobatto, Guilherme Camargo Ferraz

**Affiliations:** ^1^Laboratory of Equine Exercise Physiology and Pharmacology, Department of Animal Morphology and Physiology, School of Agricultural and Veterinarian Sciences, São Paulo State University, FCAV/UNESP, São Paulo, Brazil; ^2^Federal University of Piauí (UFPI), Campus Professora Cinobelina Elvas (CPCE), Bom Jesus, Piauí, Brazil; ^3^Laboratory of Applied Sport Physiology, School of Applied Sciences, State University of Campinas, FCA/UNICAMP, São Paulo, Brazil; ^4^Equine Sports Medicine Laboratory, Department of Veterinary Clinic and Surgery, School of Agricultural and Veterinarian Sciences, São Paulo State University, FCAV/UNESP, São Paulo, Brazil; ^5^Academic Department of Veterinary Medicine, Federal University of Rondônia, UNIR, Rolim de Moura, Rondônia, Brazil

**Keywords:** acid-base balance, cool-down, exercise, heart rate, lactate, MCT1, MCT4, whole-body vibration

## Abstract

**Introduction:**

Several studies have explored alternatives to enhance the performance, health, and safety of sports horses. One promising method involves the use of vibrating platforms (VP), which offer passive exercise stimulation via mechanical oscillations distributed throughout the body. This type of exercise is referred to as whole-body vibration (WBV) and is an emerging strategy for accelerating muscle recovery. This study examined the dynamics of proteins responsible for transporting monocarboxylates (MCT1 and MCT4), and their relationship with lactatemia and acid-base balance in connection with WBV recovery following intense treadmill exercise in horses.

**Methods:**

Eight crossbred horses underwent the standardized exercise test on the treadmill to determine the velocity corresponding to the lactate threshold. This velocity was used to prescribe the external load of the acute intense exercise bout (AIEB), which was performed to recruit rapidly fatigable type II muscle fibers and induce hyperlactatemia and metabolic acidosis. The horses were assigned to three experimental groups in a crossover design, with a 7-day washout period. The treadmill group (TG) actively recovered through low-intensity treadmill walking. The WBV group (WBVG) followed a stepwise recovery protocol on VP, with each step lasting 2 min and the frequencies decreasing in a specific order: 76, 66, 55, 46, and 32 Hz. The sham group (SG) was designated for horses with the VP turned off. All groups experienced a uniform recovery strategy duration of 10 min. Heart rate (HR), rectal temperature (RT), lactatemia, glycemia, acid-base status and electrolytes, strong ion difference (SID), and muscle monocarboxylate transporters (MCT1 and MCT4), were assessed.

**Results:**

AIEB induced positive chronotropic effects, hyperlactatemia and moderate metabolic acidosis in all experimental groups. All groups also showed transitory hyperthermia, hyperglycemia, hypernatremia, hyperchloremia, hyperkalemia and SID reduction. HR was higher in TG than in the WBVG and SG immediately after the recovery procedures. Between the groups, there was no change in RT, lactatemia, glycemia and MCT1 and MCT4 content. Regardless of groups, the MCT4 content decreased 3 and 6 h after recovery strategies.

**Discussion:**

It was concluded that a single whole-body vibration session did not enhance recovery of lactatemia or acid-base balance in horses after intense treadmill exercise.

## 1 Introduction

Improving the quality and safety of athletic performance is an important scientific topic for humans and other animal species. Equestrian associations aim to enhance the athletic ability, health and wellbeing of horses involved in sports. This field encompasses various research areas, including whole-body vibration (WBV). WBV is considered a non-invasive and non-pharmacological rehabilitation and recovery strategy, which uses vibrating platforms that produce mechanical vibrations, exposing the organism to a mechanotransduction process, and has been studied in humans (1–10) and in rodents (11–15) as a training device, to improve involuntary neuromuscular activation, biomechanics, and physiological variables related to performance (16–18).

It is vital to understand that improper use of vibration therapy can lead to adverse effects in men, including skin erythema, edema, pain (19), hematuria (20), knee discomfort (21, 22), and fatigue (21). Furthermore, research has shown progressive intervertebral disc degeneration in mice (23) and worsened chronic lameness in horses after extended WBV sessions (24) and intensified thoracolumbar pain with short-term use (25). Customizing vibration parameters such as amplitude, frequency, and oscillation magnitude is essential, as individual responses vary (16).

Recent studies demonstrate the benefits of WBV in addressing obesity and enhancing physical performance. One study found that WBV decreased lactate, ammonia, and serum creatine kinase activity, while increasing blood glucose in rats subjected to swimming and WBV, indicating its potential to alleviate fatigue (11). Another study on middle-aged mice revealed that WBV improved aerobic fitness and reduced fatigue (12). Research on combining dehydroepiandrosterone supplementation with WBV in mice showed remarkable results, including enhanced exercise performance, increased serum testosterone and blood glucose, and higher liver and muscle glycogen (14). These findings further emphasize WBV's role in boosting fitness and overall wellbeing.

Isometric squat training for healthy active males on a vibrating platform effectively increases time to exhaustion and reduces neuromuscular fatigue in knee extensor muscles (8). Research indicates that combining physical exercise with whole-body vibration (WBV) lowers lactate levels, heart rate (HR), and blood pressure (2, 4, 5). However, a study on recreational runners using WBV and drop jumps as warm-ups found no significant changes in ankle dorsiflexion or running times (10). Similarly, a study on horses assessed the effects of WBV as a warm-up strategy on various physiological variables and found no significant results (26), underscoring the complexity of WBV's effectiveness in different contexts.

Methods to restore homeostasis after intense exercise, particularly in terms of body temperature recovery, hyperlactatemia, and metabolic acidosis, are gaining attention in equine studies (27–33). In the horse industry, WBV is proposed as a technique to accelerate recovery post-exercise. While manufacturers endorse this method (34), its use in equestrian centers often lacks scientific evidence, at least to our knowledge. We hypothesize that WBV enhances lactate clearance through monocarboxylate transporters, facilitating the transport of L-lactate across the sarcolemma and restoring acid-base balance after vigorous exertion. It should be highlighted that this paper seeks to improve the abilities of veterinarians, researchers, and horse owners in maintaining horses' health and wellbeing by bridging the gap between experimental research on WBV and real-world applications.

## 2 Materials and methods

The study was approved by the Ethics Committee on the Use of Animals (CEUA) of the School of Agricultural and Veterinarian Sciences UNESP-Jaboticabal Campus (protocol no. 08373/19) and carried out during October and November 2022 at GPS coordinates-21.245408 latitude and−48.299415 longitude.

### 2.1 Horses

Eight crossbreed horses were used (one gelding and seven females), with an average body mass of 406 ± 33 kg and aged between 7 and 19 years. The horses belonged to the didactic herd of the Equine Exercise Physiology and Pharmacology Laboratory (LAFEQ), Department of Animal Morphology and Physiology, School of Agricultural and Veterinarian Sciences, São Paulo State University (FCAV/UNESP), Jaboticabal, São Paulo, Brazil. The horses were included in the study after ~2 months of adaptation to the experimental conditions, such as handling and feeding, treadmill (Galloper G 5500, Sahinco, Palmital, SP) and vibrating platform (TheraPlate^®^, Weatherford, TX, USA). The horses were kept in paddocks and fed with Tifton 85 hay, in addition to 0.2% of body mass in concentrate once a day. Mineralized salt and water were provided *ad libitum*. They were regularly hoofed, and, during the experimental period, they did not use horseshoes. Before the start of the experimental stages, the horses underwent clinical and hematological examinations to determine their healthiness and were previously vaccinated and treated with anthelmintics.

### 2.2 Determination of the velocity corresponding to the lactate threshold (VLT)

A standardized exercise test (SET) was conducted on all horses to determine the velocity corresponding to the lactate threshold (VLT), which was used to prescribe the external load for an acute intense exercise bout (AIEB). The SET protocol was adapted from Lamprecht and Williams (35) and performed on a treadmill. The warming-up consisted of 3 min of walking at a speed of 1.5 m/s with a surface inclination of 0%, followed by 2 min of trotting at 2.5 m/s and 5% positive slope. The SET started at 4.0 m/s, with the speed increasing by 1.0 m/s at 2-min intervals. The horses underwent a 2-min active recovery period at 1.5 m/s between each speed step. A surface inclination of 5% was maintained throughout the incremental phase. To ensure the horses' safety, the SET concluded when the horses reached a heart rate of 200 bpm or at the end of the speed stage of 9 m/s. At the end of the SET, the horses cooled down by walking at a comfortable speed for everyone, with an inclination of 0% (Supplementary Table S1).

Two complementary methods were used to determine the velocities corresponding to the lactate threshold: visual (VLT_V_) and bi-segmented (VLT_BI_). During the SET, the inflection point of the lactate-velocity curve was determined by analyzing the plasma lactate concentrations of each horse, indicating a non-linear increase. Four exercise physiology experts established this point. They then associated this point with the corresponding speed (x-*axis*) to establish the velocity corresponding to the visual lactate threshold (VLT_V_). This method was refined by applying bi-segmented linear regression (VLT_BI_). Two regression lines from the ordinary least squares regression were used, and the VLT_V_ obtained was considered. By equating the values of the two equations related to the y-values, the speed (x) was obtained. To ensure that the external load of AIEB would recruit rapidly fatigable type II muscle fibers and induce physiologic hyperlactatemia and metabolic acidosis, the highest speed obtained between VLT_V_ and VLT_BI_ for everyone was used, and the values are presented in Supplementary Table S2.

### 2.3 Acute intense exercise bout (AIEB)

All horses performed AIEB with an external load above the VLT. Initially, the horses were subjected to a warming-up period of 1 min at a speed of 1.5 m/s and 0% inclination, followed by 2 min at a speed of 3.5 m/s and 2 min at VLT, both with 5% inclination. To intensify the external load, aiming to induce hyperlactatemia and metabolic acidosis, the AIEB was completed in the gallop, with 2 min at 110% of the VLT and 3 min at 130% of the VLT, with an inclination of 5%. Once the AIEB was finished, the recovery phase commenced to assess the suggested strategies for recovery.

### 2.4 Experimental groups

Before the experiment began, the horses were randomly assigned into three experimental groups, in a crossover design, each specifically designed to evaluate a different type of recovery after AIEB. All horses participated in all three experimental groups, carried out in three blocks. The randomization process involved numbering the animals from 1 to 8, followed by a drawing to create the experimental blocks (1, 2, and 3) using an Excel spreadsheet with the formula =RANDBETWEEN(1;8). All strategies for recovery were meticulously planned and executed, each lasting 10 min. The treadmill group (TG) performed the active recovery period on the treadmill at a comfortable speed for each horse (between 1.2 and 1.6 m/s). The horses in the sham group (SG) remained on the turned-off vibrating platform, providing a control group for the experiment. The whole-body vibration group (WBVG) performed one WBV session during recovery. The horses in the SG and WBVG groups began their recovery roughly 5 min after the end of AIEB. For the TG group, the horses waited 5 min on the treadmill before the active recovery period began, further ensuring the scientific rigor of the experiment.

### 2.5 Acute whole-body vibration (WBV) session

The horses in the WBVG group underwent a WBV session after AIEB, using the Theraplate^®^ (Original Equine Unit Model K21, Theraplate, Weatherford, TX, USA). This VP, equipped with a unique “proprietary technology” called vortex wave stimulation, utilizes centrifugal force and internal oscillating movement to provide zero-impact therapy. The horses were maintained in a quadrupedal position on the VP. The WBV recovery protocol, developed according to the manufacturer's guidelines from TheraPlate (TPR, LLC), was implemented for horse recovery following AIEB. This protocol lasted 10 min and was designed to gradually decrease the frequency levels. It included 2 min at each load corresponding to 100%, 80%, 60%, 40%, and 20% of the equipment's maximum motor capacity, which correspond to 76 Hz [peak displacement (Dpeak) = 0.09 mm; peak acceleration (Apeak) = 10.36 m/s^2^], 66 Hz (Dpeak = 0.12 mm; Apeak = 9.96 m/s^2^), 55 Hz (Dpeak = 0.16 mm; Apeak = 9.46 m/s^2^), 46 Hz (Dpeak = 0.22 mm; Apeak = 9.12 m/s^2^), and 32 Hz (Dpeak = 0.51 mm; Apeak = 10.35 m/s^2^), respectively. The frequency and peak acceleration were measured using an accelerometer (App Accelerometer meter). The peak displacement was calculated using the formula:


(1) $$\begin{array}{l} Dpeak=\left[\frac{Apeak}{2\times \pi^{2}\times frequency^{2}}\right]\times 1000. \end{array}$$

### 2.6 Assessment methods

#### 2.6.1 Heart rate (HR)

HR was measured using a Polar Equine Heart Rate Monitor for Trotters (Polar Electro, Kempele, Finland). The peak HR for each stage was extracted from the Polar Flow^®^ app (https://flow.polar.com/). The data were collected before (baseline) and after (A) AIEB, before (BR) and after (AR) recovery, and 10 min at the end of the recovery period (10 min).

#### 2.6.2 Rectal temperature (RT)

A digital predictive thermometer (Clean View RM-TD0403A, Relaxmedic, Vargem Grande Paulista, Brazil) was used to measure RT. Temperature readings were taken at three time points: baseline, A, and AR.

#### 2.6.3 Lactatemia and glycemia

Blood samples were collected through jugular vein catheterization in tubes containing sodium fluoride and EDTA to determine the plasma concentration of lactate and glucose. The samples were centrifuged, and the plasma was separated and immediately analyzed. The analyses were carried out using the electroenzymatic method with an automatic bioanalyzer (YSI 2300 Stat Plus^®^, Ohio-USA). Samples were obtained at the following time points: baseline, A, BR, AR, 10 min, and 1 h after the end of the recovery period.

#### 2.6.4 Blood gases and electrolytes

Blood was collected through jugular vein catheterization to analyze blood gases and electrolytes, avoiding contact with environmental oxygen and carbon dioxide, in 3.0 mL blood gas syringes. Samples were collected at three-time points: baseline, A, and AR. Analyses were performed immediately after obtaining the samples using a portable analyzer (i-STAT Analyzer, Abbott Laboratories, Libertyville Township, IL, USA), cartridge EC8+, which contains tests for electrolytes, chemistries, blood gases, hematocrit, and hemoglobin. The variables measured were pH, venous partial pressure of carbon dioxide (P_V_CO_2_), total carbon dioxide (tCO_2_), base excess/deficit (BE_ecf_), anion gap (AnGap), bicarbonate (${HCO}_{3}^{-}$), sodium ion (Na^+^), potassium ion (K^+^), chloride ion (Cl^−^), total hemoglobin (Hb), hematocrit (Hct) and urea nitrogen (Ure). pH and P_V_CO_2_ values were adjusted for rectal temperature (36, 37). The strong ion difference (SID) is calculated as the difference between the total concentration of strong cations and the total concentration of strong anions, using the formula:


(2) $$\begin{array}{l} SID=\left(\left[Na^{+}\right]+\left[K^{+}\right]\right)-\left(\left[Cl^{-}\right]+\left[Lac^{-}\;\right]\right). \end{array}$$

#### 2.6.5 Muscle samples

The horses were subjected to muscle biopsies at the following time points: baseline (around 18 h before AIEB), AR, 3 and 6 h after recovery, as described previously (38). The horses were restrained in a quadrupedal position on a horse stock before biopsy sampling. The needle insertion site in the gluteus medius (gluteal) was in the middle third between the coxal tuberosity and the tailhead. Collections were alternated between the right and left sides of the gluteus medius muscles. The sample collection site was shaved and cleaned with chlorhexidine before being rinsed with 70% ethanol. A local anesthetic block was then applied with subcutaneous infiltration of 3 mL of 2% lidocaine hydrochloride without vasoconstrictor. After 5 min, an incision was made in the skin, subcutaneous tissue and gluteal fascia at the needle insertion site using a sterile disposable #24 scalpel blade. Next, the sterile 6.0-mm Bergström-type needle was introduced into the previously made incision to a depth of 60 mm at a 90° angle to obtain the muscle fragments. The muscle samples were immediately snap-frozen in liquid nitrogen and later stored in a −80°C freezer until the analysis.

##### 2.6.5.1 Biomolecular analyzes of monocarboxylate transporters 1 (MCT1) and 4 (MCT4)

The analyses were performed based on a previous study in mice (39).

###### 2.6.5.1.1 Extraction and quantification of total proteins

The samples (25 mg) were homogenized, through maceration, in 300 μL of RIPA buffer with the following components: 1% Tris HCl (50 mM), NaCl (150 mM), EDTA (1 mM), IGEPAL^®^ CA-630 (1%), Deoxycholate (0.5%), SDS (0.1%), 1% protease inhibitor (Protease and Phosphatase Inhibitor Cocktail, cat# P8340, Sigma Aldrich^®^) and 1% phosphatase inhibitor (Phosphatase II Inhibitor Cocktail Set, cat# US1524625-1SET, Calbiochem^®^, San Diego, CA, USA), ensuring the highest quality of protein preservation. Then, the samples were placed in a sonicator (Q55 Sonicator, Qsonica^®^, Newtown, CT, USA) twice for 5 s (60%). Subsequently, the lysates were centrifuged at 12,000 rpm for 10 min at 4°C (Centrifuge 5424 R, Eppendorf AG, Hamburg, Germany). Soon after, the supernatant was separated (total protein extract), and the pellet was discarded. The Bradford colorimetric method was used to quantify total proteins. The absorbance was read at a wavelength of 595 nm using a spectrophotometer.

###### 2.6.5.1.2 Electrophoresis

The samples (40 μg of total protein) were mixed with LDS buffer (lithium dodecyl sulfate and 1% mercaptoethanol) and taken to a dry bath at 94°C for 10 min. Then, the samples were placed on ice for 5 min. Next, 9 μL of each sample was pipetted into the gel wells (Mini Protean TGX Precast Protein Gels, 10% 15-well, Bio-Rad^®^), and then 7.5 μL of weight molecular marker was pipetted in the first well of the gel. Electrophoresis was performed using specific equipment (PowerPac 300 Electrophoresis Power Supply—Bio Rad, São Paulo, SP, Brazil) at a constant 130 V for ~60 min. An iBlot™ 2 Gel Dry Transfer Device (20 V for 7 min cat# IB21001, Waltham, MA, USA) was used to transfer proteins (PVDF) to the membrane (Invitrogen™, iBlot™ 2 Transfer Stacks cat# IB # IB24002, Waltham, MA, USA).

###### 2.6.5.1.3 Western blotting of MCT1 and MCT4 proteins

Membranes were subjected to fluorescence staining (RevertTM total protein stain, cat # 926-11010) and scanned using the 700 nm channel of the LI-COR Odyssey Fc imaging system. Afterwards, blocking was performed in 1% milk (skimmed milk powder, cat# 9999, Cell Signaling Technology^®^, Danvers, MA, USA) in PBS (10 mL of PBS, 0.1 g of NFDM milk) for 1 h on an orbital shaker. Then, the membranes were immediately incubated with 5% milk in PBS-Tween for 1 h at room temperature (for MCT4 antibody, cat #BS-2698R, Bioss, Woburn, MA, USA) or overnight at 4°C (for MCT1 antibody, cat #20139-I-AP, Proteintech, Rosemont, IL, USA). Antibody binding was detected by preabsorbed goat anti-rabbit IgG H&L (cat. #ab216773) (IRDye 800CW) at a dilution of 1:20,000 for 1 h at room temperature in the dark. Fluorescence was detected at 800 nm using the same imaging system (Odyssey Fc, LI-COR Biosciences, Lincoln, NE, USA). For accurate results, protein expression was normalized by dividing the antibody signal by the band normalization factor.

### 2.7 Statistical analysis

The experiment was designed with a 3 × 4 factorial design, with 3 treatments (TG, WBVG, SG) and 4 timepoints (baseline, AR, 3 h, 6 h). It included three paired replications and a split-plot scheme. We used a paired randomized crossover design, allowing horses to experience three recovery strategies, each followed by a vital 7-day washout period. Muscle biopsy assessments were used for the proposed model. Statistical analysis was conducted using RStudio software for Windows (version 2023.06.0+421 “Mountain Hydrangea”), which uses the format “function {package}” to present functions and packages. Normality of residuals was assessed using the Shapiro-Wilk test (shapiro_test {rstatix}) and QQ graphs (qqplot {stats}). The Levene test (leveneTest {car}), considered to be more robust against potential deviations from normality, was applied to evaluate the homoscedasticity of variances. A two way ANOVA for repeated measures (anova_test {rstatix}) and paired *t*-tests with Bonferroni correction (pairwise_*t*_test {rstatix} with argument “p.adjust.method = bonferroni”) were used for statistical analysis. To evaluate potential improvements in aerobic fitness throughout the experiment, we assessed aerobic fitness between blocks (periods) using one way ANOVA for repeated measures (anova_test {rstatix}), followed by *post-hoc* paired *t*-tests. We applied the Bonferroni correction (pairwise_*t*_test {rstatix} with argument “p.adjust.method = bonferroni”) to reduce the risk of false-positive results (type I error) in our heart rate and plasma lactate measurements. The use of Bonferroni correction is warranted due to the large number of comparisons made after each ANOVA. This method ensures that the significance level remains consistent across all comparisons (40). The area under the curve (AUC) (auc {MESS}) for plasma lactate, MCT1 and MCT4 was determined using all samples obtained by the trapezoidal method for numerical integration (41). The presence of linear relationships between the variables was evaluated by calculating the Pearson correlation coefficient (*r*) (cor {stats}). Based on the value obtained, the following degrees of correlation were defined as *r* = 0, absence of correlation; *r* < ±0.29, weak correlation; ±0.3 < *r* < ±0.49, moderate correlation; ±0.5 < *r* < ±0.79, strong correlation; *r* > ±0.8, very strong correlation. We used a significant level of 5% for all analysis.

## 3 Results

### 3.1 Aerobic conditioning between experimental blocks

**Figure 1:**
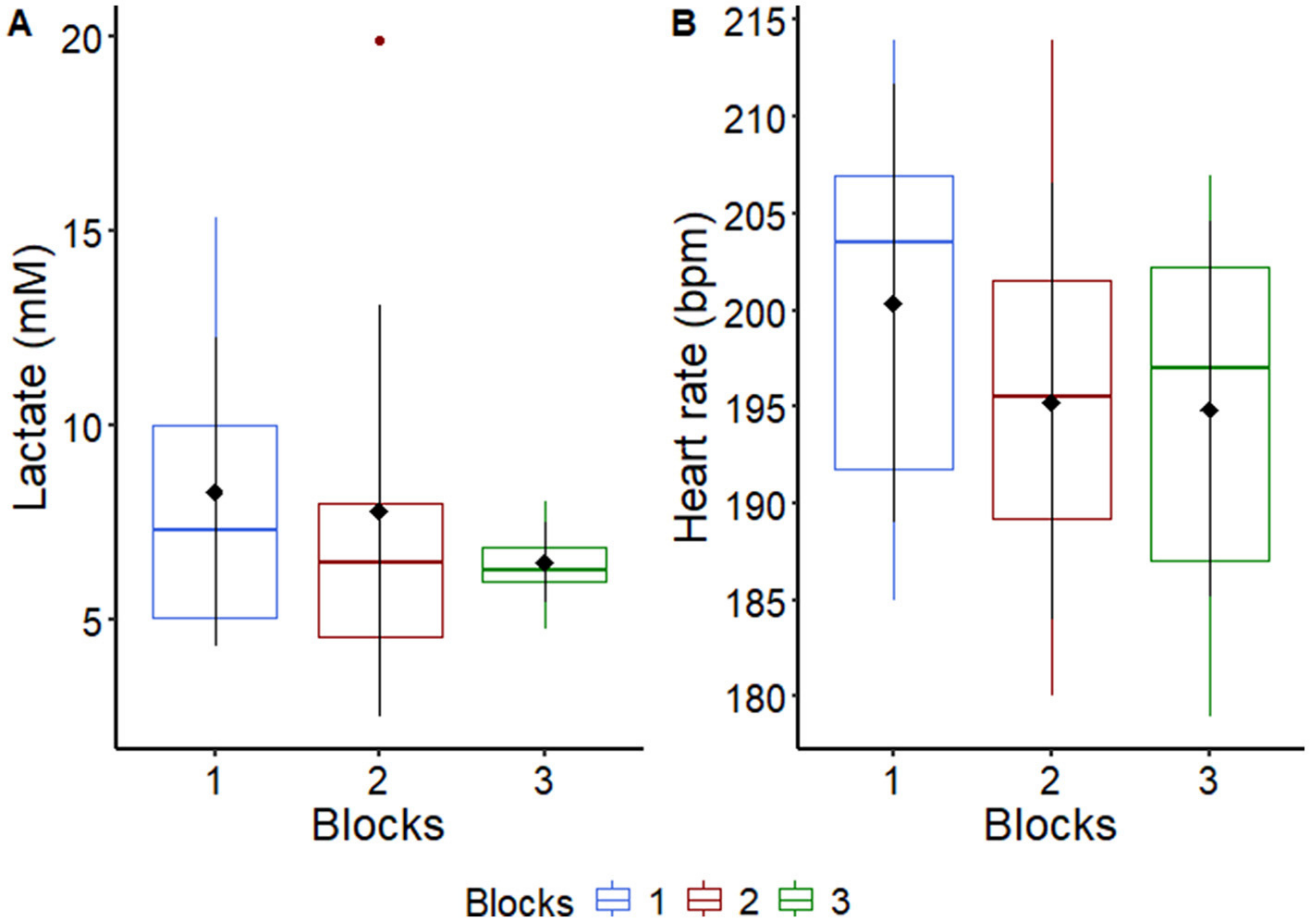
Graphical representation of median, interquartile range and means ± standard deviation (pointrange) of **(A)** plasma lactate and **(B)** heart rate of horses (*n* = 8) after an acute intense exercise bout in each experimental block. For more details see Supplementary Table S3.

Aiming to verify a possible increase in aerobic fitness throughout the experiment, the variables heart rate and plasma lactate were checked, right after the end of AIEB, and there was no difference between blocks (*P* = 0.114, for HR and *P* = 0.36, for lactate), indicating that there was no gain in physical conditioning throughout the experimental trial course (Figure 1; Supplementary Table S3). It is worth noting that all horses used in the current study were previously conditioned. No horses were interrupted during the test attributable to fatigue, as indicated by their ability to maintain speed on the treadmill and preserve motor coordination.

### 3.2 Heart rate

**Figure 2:**
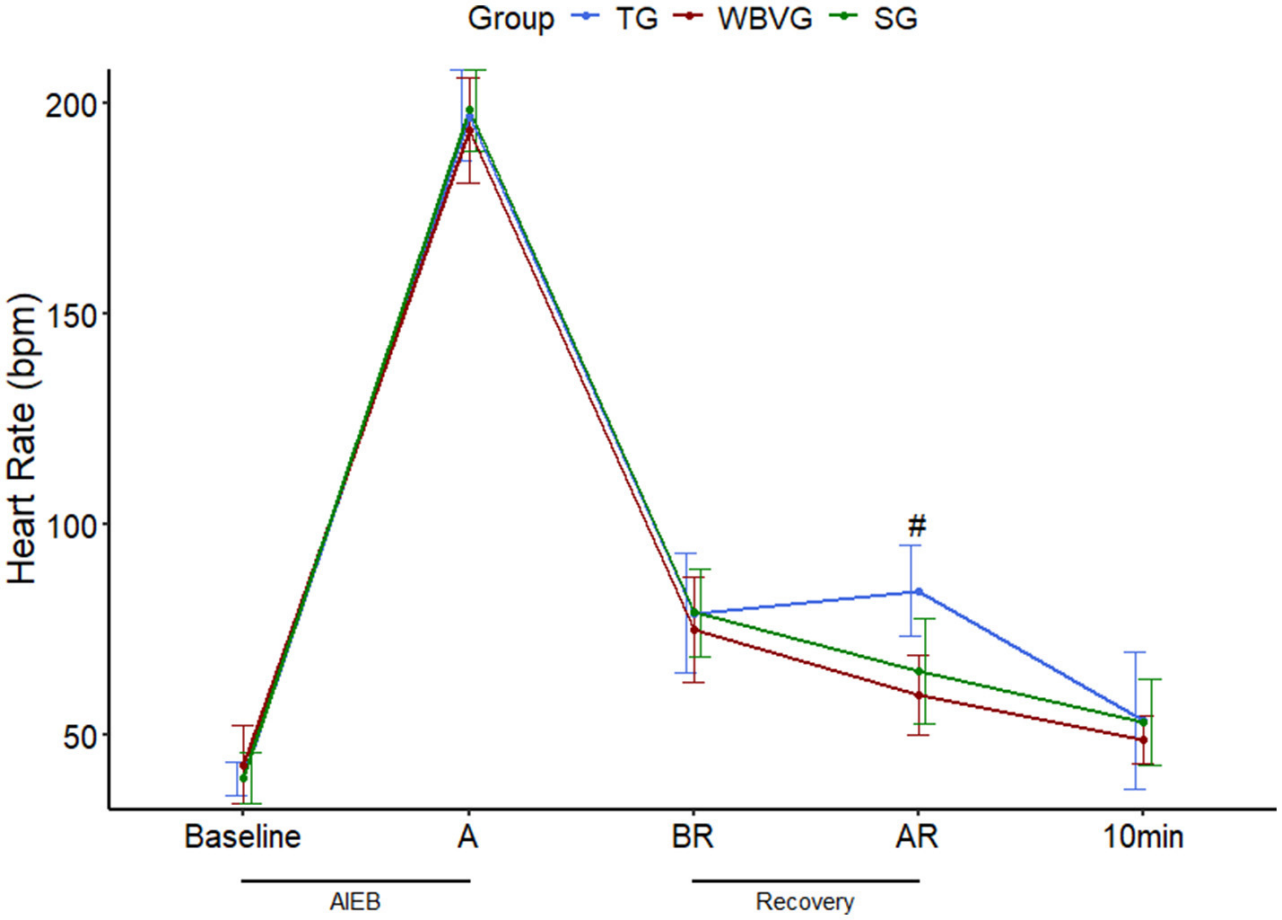
Graphical representation of means ± standard deviation of heart rate of horses (*n* = 8) submitted to an acute intense exercise bout (AIEB) and a 10-min recovery period on the treadmill (TG), on a whole-body vibration session (WBVG), or the vibrating platform off (SG). ^#^Indicates that HR was higher in TG concerning SG and WBVG at a significance level of *P* < 0.05. A, after AIEB; BR, before recovery; AR, after recovery; 10 min, 10 min after the end of the recovery period. For more details see Supplementary Table S4.

The HR average values are shown in Supplementary Table S4. As expected, AIEB induced positive chronotropic responses in all experimental groups. After implementing recovery strategies, the HR was higher (*P* = 0.001) in the TG compared with the other groups (Figure 2).

### 3.3 Rectal temperature

**Figure 3:**
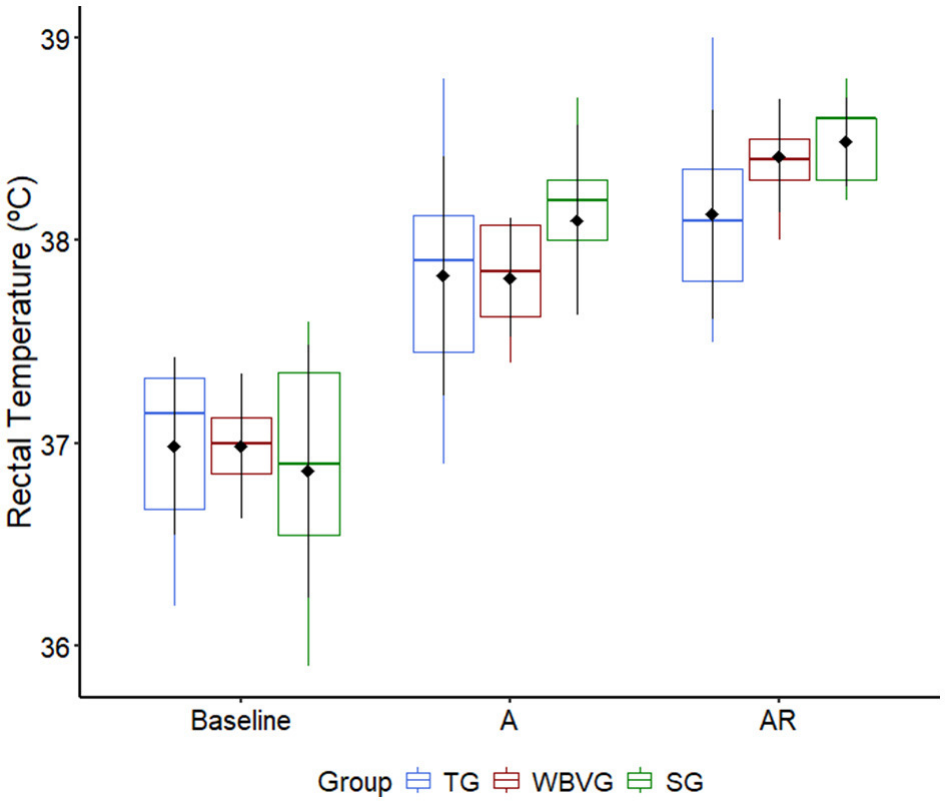
Graphical representation of median, interquartile range and means ± standard deviation (pointrange) of the rectal temperature of horses (*n* = 8) submitted to an acute intense exercise bout (AIEB) and a 10-min recovery period on the treadmill (TG), on a whole-body vibration session (WBVG), or the vibrating platform off (SG). A, after AIEB; BR, before recovery; AR, after recovery. For more details see Supplementary Table S5.

The RT of the animals in all experimental groups increased after AIEB (*P* = 0.037; Supplementary Table S5). There was no difference between groups at any time point (*P* = 0.876; Figure 3; Supplementary Table S5).

### 3.4 Lactatemia and glycemia

**Figure 4:**
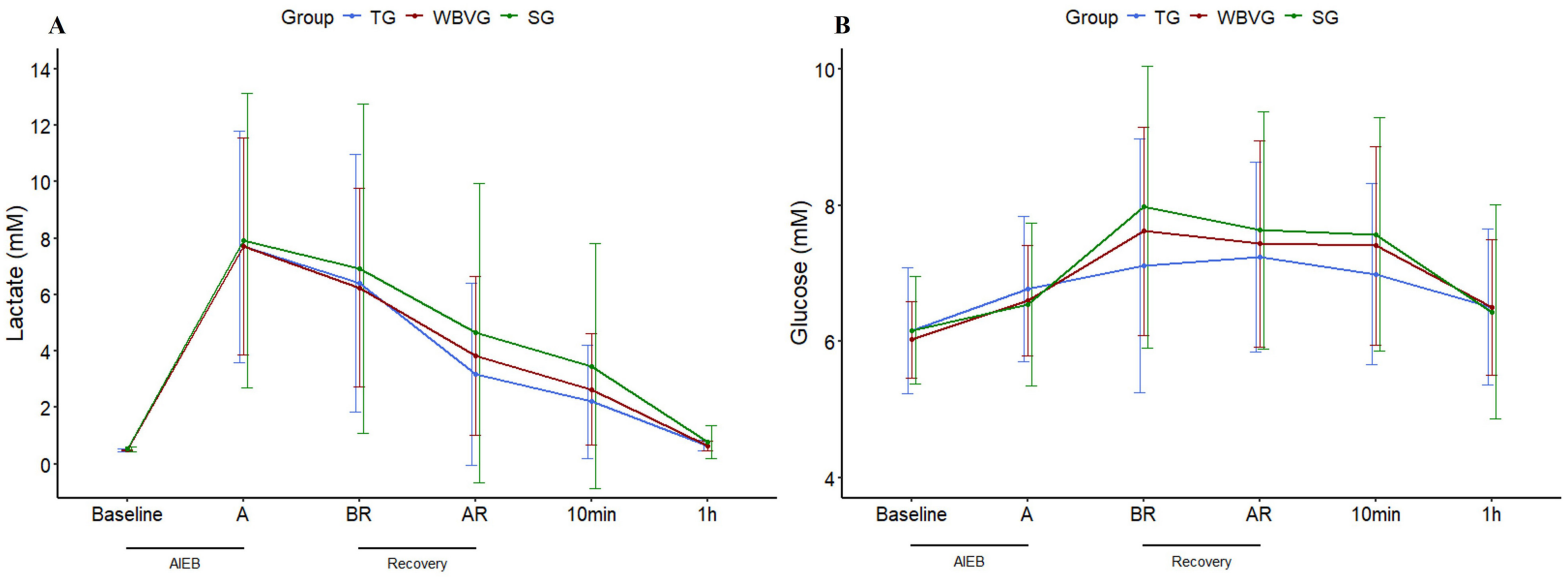
Graphical representation of means ± standard deviation of **(A)** plasma lactate and **(B)** plasma glucose of horses (*n* = 8) submitted to an acute intense exercise bout (AIEB) and a 10-min recovery period on the treadmill (TG), on a whole-body vibration session (WBVG), or the vibrating platform off (SG). A, after AIEB; BR, before recovery; AR, after recovery; 10 min, 10 min after the end of the recovery period. For more details see Supplementary Table S6.

Although there was a trend toward an increase in lactatemia (*P* = 0.08) for SG, there was clearly physiological hyperlactatemia in all groups immediately after AIEB, which decreased over time, regardless of recovery strategy (Figure 4A; Supplementary Table S6). It should be emphasized that there is no difference between groups at any time for glycemia (*P* = 0.488) and lactatemia (*P* = 0.352; Figure 4; Supplementary Table S6). Plasma glucose peaked five (BR) to 15 min (AR) after AIEB, returning to baseline values 1h after all recovery strategies (Figure 4B; Supplementary Table S6).

### 3.5 Blood gases and electrolytes

Except for PvCO_2_ and Ure, all blood gas variables showed significant changes after AIEB for all experimental groups, indicating metabolic acidosis (Table 1). Between the groups, a higher Ure was identified in the WBVG in relation to the TG at baseline (*P* = 0.01). It is worth noting that the average values of Ure remained within the reference range for the equine species (42).

**Table 1:** Means ± standard deviation of blood gases and electrolytes of horses submitted to an acute intense exercise bout (AIEB) and a 10-minute recovery period on the treadmill (TG), on a whole-body vibration session (WBVG), or the vibrating platform off (SG).

**Variable**	**TG**			**WBVG**			**SG**		
	**Baseline**	**A**	**AR**	**Baseline**	**A**	**AR**	**Baseline**	**A**	**AR**
pH	7.43 ± 0.02^a^	7.33 ± 0.06^b^	7.41 ± 0.04^c^	7.42 ± 0.02^a^	7.33 ± 0.07^b^	7.38 ± 0.04^c^	7.42 ± 0.02^a^	7.32 ± 0.08^b^	7.36 ± 0.08^c^
PvCO_2_	43.1 ± 2.5	45.5 ± 4.4	39.1 ± 3.9	44.8 ± 2.8	44.2 ± 4.8	42.8 ± 4.1	42.4 ± 3.8	43.7 ± 3.9	42.8 ± 5.7
tCO_2_	29.6 ± 2.1^a^	25.0 ± 2.4^b^	25.2 ± 4.5^b^	30.6 ± 1.9^a^	24.0 ± 3.1^b^	26.2 ± 3.5^b^	29.0 ± 3.0^a^	23.8 ± 4.2^b^	25.4 ± 5.5^b^
BE_ecf_	3.88 ± 2.03^a^	− 1.88 ± 3.27^b^	− 0.38 ± 5.15^c^	5.12 ± 1.88^a^	− 2.62 ± 3.66^b^	0.63 ± 3.78^c^	3.25 ± 2.82^a^	− 3.00 ± 5.18^b^	− 1.12 ± 6.49^c^
AnGap	12.1 ± 1.5^a^	17.1 ± 2.6^b^	16.0 ± 3.3^c^	11.4 ± 1.2^a^	17.9 ± 3.6^b^	14.5 ± 2.8^c^	11.3 ± 1.9^a^	16.5 ± 2.3^b^	15.6 ± 4.5^c^
${\text{HCO}}_{3}^{-}$	28.4 ± 1.9^a^	23.8 ± 2.5^b^	24.2 ± 4.6^c^	29.4 ± 1.8^a^	22.9 ± 3.0^b^	25.2 ± 3.3^c^	27.8 ± 2.8^a^	22.6 ± 4.1^b^	24.1 ± 5.3^c^
Na^+^	139 ± 1^a^	141 ± 2^b^	139 ± 3^a^	138 ± 1^a^	140 ± 2^b^	137 ± 1^a^	138 ± 1^a^	140 ± 2^b^	138 ± 1^a^
K^+^	3.59 ± 0.23^a^	4.90 ± 0.67^b^	3.80 ± 0.43^c^	3.58 ± 0.21^a^	5.05 ± 0.48^b^	3.69 ± 0.17^c^	3.62 ± 0.14^a^	5.09 ± 0.32^b^	3.76 ± 0.18^c^
Cl^−^	102 ± 1^a^	105 ± 2^b^	103 ± 4^a^	101 ± 2^a^	105 ± 2^b^	101 ± 2^a^	102 ± 2^a^	106 ± 3^b^	102 ± 2^a^
Hb	11.6 ± 0.8^a^	16.9 ± 1.5^b^	12.6 ± 1.3^c^	11.6 ± 0.6^a^	17.1 ± 1.7^b^	12.9 ± 1.3^c^	11.5 ± 0.9^a^	17.4 ± 1.3^b^	13.2 ± 1.3^c^
Hct	34.1 ± 2.2^a^	49.6 ± 4.2^b^	37.1 ± 3.8^c^	34.2 ± 1.8^a^	50.2 ± 5.1^b^	38.0 ± 3.8^c^	34.1 ± 2.5^a^	51.2 ± 3.8^b^	39.0 ± 3.9^c^
Ure	17.6 ± 2.6^Aa^	18.5 ± 2.7^b^	17.2 ± 2.7^ab^	19.6 ± 1.7^B^	19.9 ± 2.7	19.1 ± 2.6	17.8 ± 2.6^AB^	18.8 ± 2.3	18.1 ± 2.4
SID	40.3 ± 1.8^a^	33.3 ± 4.3^b^	37.1 ± 4.5^c^	40.3 ± 1.7^a^	32.8 ± 3.5^b^	35.9 ± 3.5^c^	39.2 ± 1.2^a^	31.9 ± 5.1^b^	34.9 ± 6.0^c^

A, after AIEB. AR, after recovery. PvCO_2_, venous partial pressure of carbon dioxide, in mmHg. tCO_2_, total carbon dioxide, in mM. BE_ecf_, base excess/deficit, in mM. AnGap, Anion gap, in mM. ${\text{HCO}}_{3}^{-}$, bicarbonate, in mM. Na^+^, sodium ion, in mEq/L. K^+^, potassium ion, in mEq/L. Cl^−^, chloride ion, in mM. Hb, total hemoglobin, in g/dL. Hct, hematocrit, in %. Ure, blood urea nitrogen, in mg/dL. SID, strong ion difference, in mM. A, after AIEB; AR, after recovery.

Different lowercase letters indicate differences in the intragroup comparison at *P* < 0.05.

Different capital letters indicate differences in the intergroup comparison at *P* < 0.05.

### 3.6 Biomolecular analyzes of monocarboxylate transporters 1 (MCT1) and 4 (MCT4)

**Figure 5:**
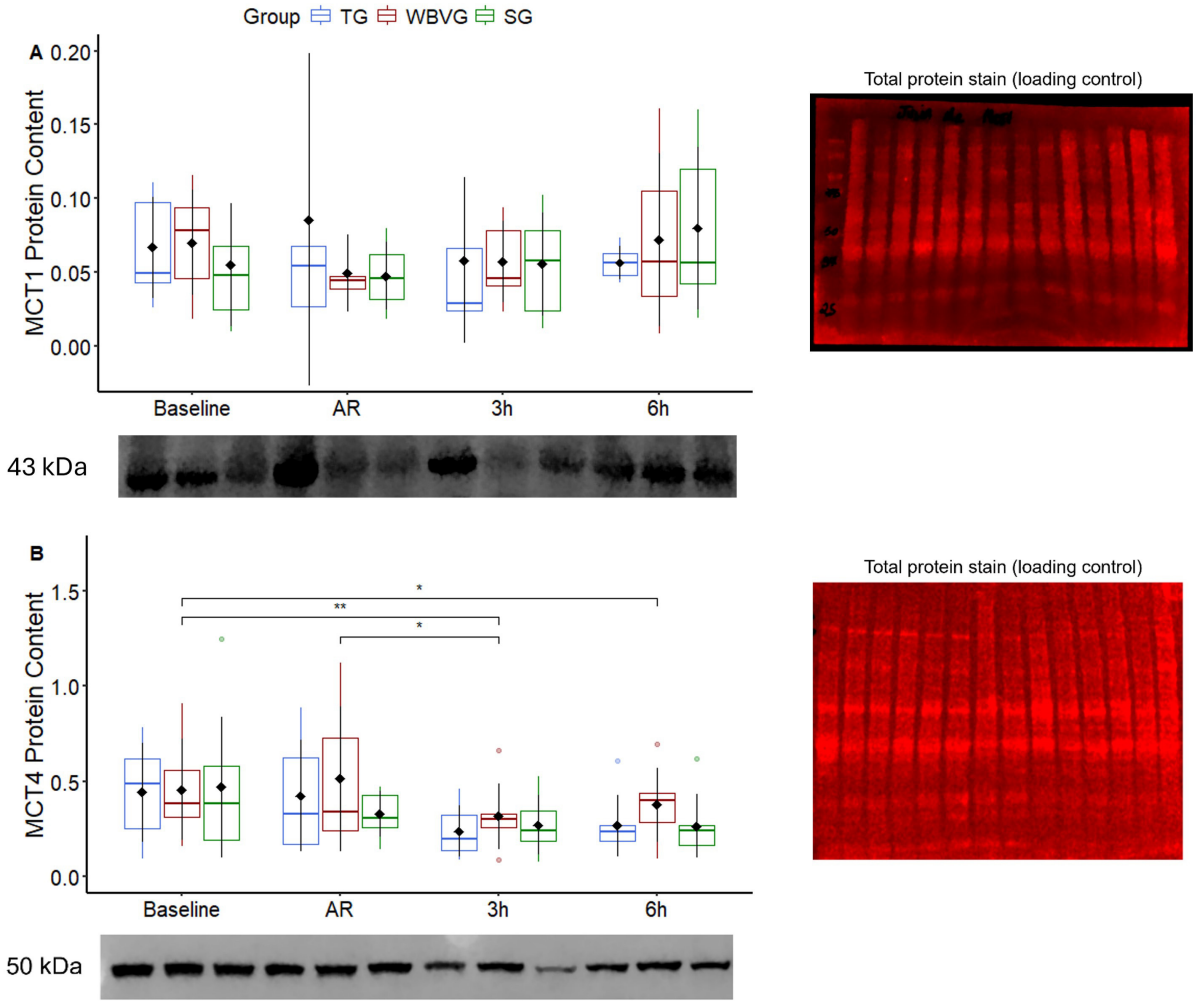
Graphical representation of median, interquartile range and means ± standard deviation (pointrange) of the **(A)** MCT1 protein content and **(B)** MCT4 protein content in the gluteal muscle of horses (*n* = 8) submitted to an acute intense exercise bout (AIEB) and a recovery period on the treadmill (TG), on a whole-body vibration session (WBVG), or on the vibrating platform off (SG). Representative images of the membranes with protein bands are shown. All membranes were stained for total protein to normalize differences between protein labeling bands. *Indicates difference between moments (*P* < 0.05). **Indicates difference between moments (*P* < 0.01). A, after AIEB; BR, before recovery; AR, after recovery; 3 h, 3 h after the end of recovery; 6 h, 6 h after the end of recovery.

There was no difference between groups (*P* = 0.705) or overtime (*P* = 0.485) for MCT1 (Figure 5A; Supplementary Table S7). No difference between groups at any time (*P* = 0.298) was detected for MCT4. There was a decrease in MCT4 protein content 3 and 6 h after recovery for all experimental groups (*P* = 0.007; Figure 5B; Supplementary Table S7).

### 3.7 Area under the curve and correlation of lactate, MCT1 and MCT4

**Figure 6:**
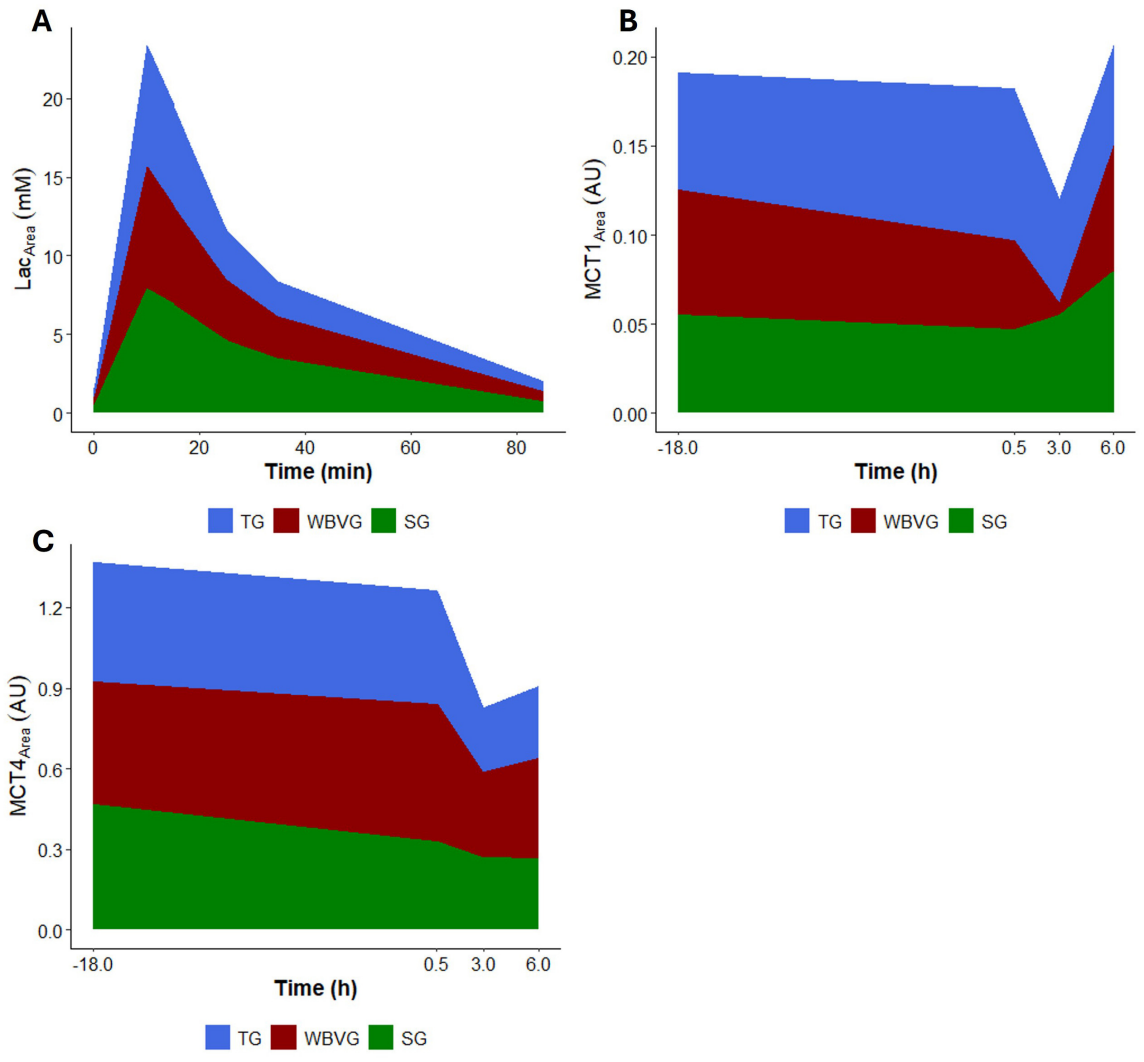
Graphical representation of area under the curve (AUC) of **(A)** plasma lactate, **(B)** MCT1 protein content and **(C)** MCT4 protein content of horses (*n* = 8) submitted to an acute intense exercise bout (AIEB) and a 10-min recovery period on the treadmill (TG), on a whole-body vibration session (WBVG), or the vibrating platform off (SG).

**Figure 7:**
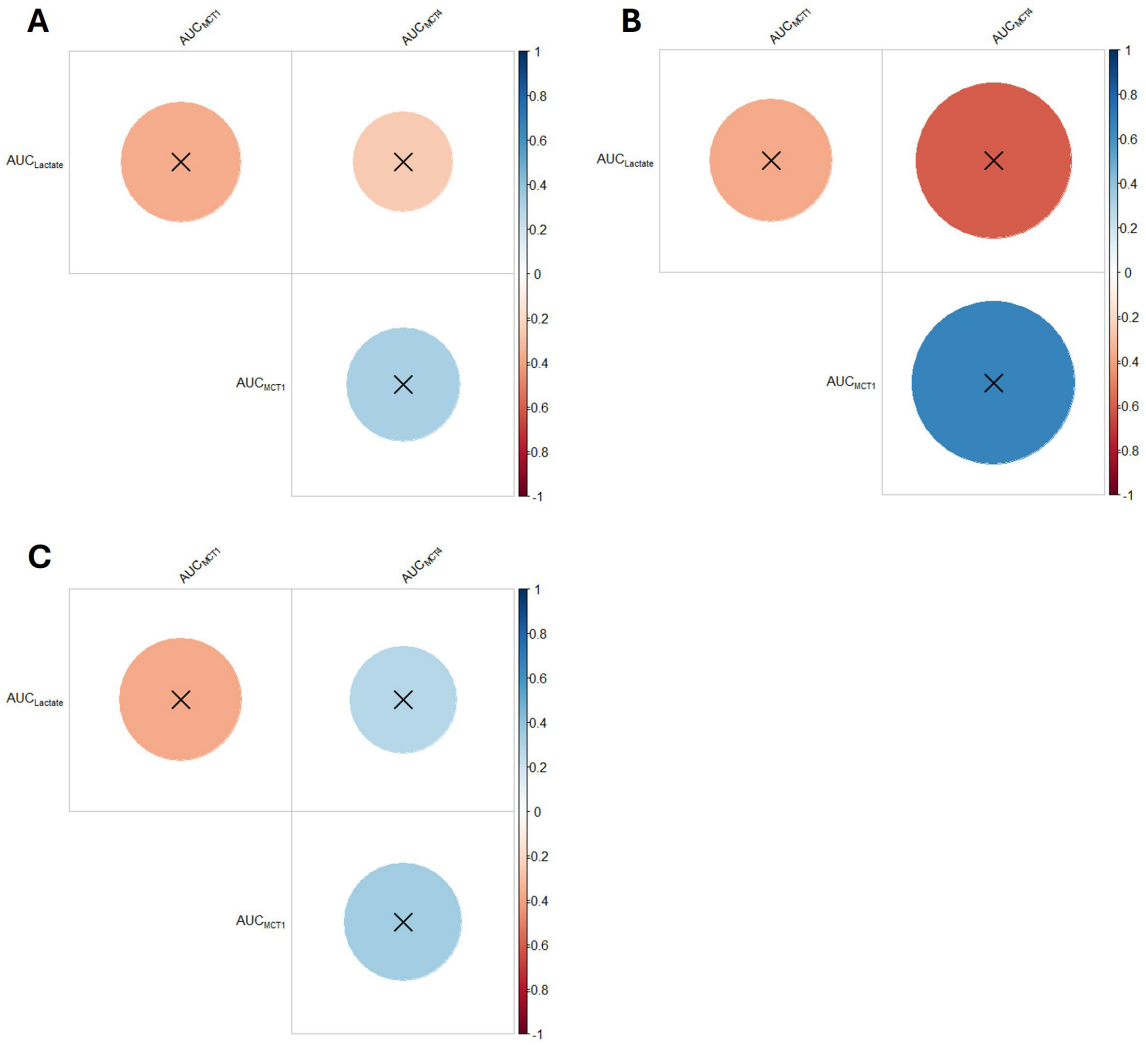
Correlogram of plasma lactate, MCT1 protein content and MCT4 protein content of horses (*n* = 8) submitted to an acute intense exercise bout (AIEB) and a 10-min recovery period on the **(A)** vibrating platform off (SG), **(B)** treadmill (TG), or **(C)** on a whole-body vibration session (WBVG).

None of the AUCs showed any differences between groups for any variable (Figure 6; Supplementary Table S8). Likewise, no correlation was significant (Figure 7; Supplementary Table S8).

## 4 Discussion

The importance of researching WBV's role in aiding horses to regain internal homeostasis should be emphasized. The current study is paving the way to identify rational therapeutic targets for WBV in horses. Contrary to our hypothesis, the results obtained regarding recovery from exercise-induced hyperlactatemia and acidosis using the WBV were not different from those horses walking on the treadmill or those of the horses placed on the VP turned off. Also, this research sheds light on the relationship between WBV, horses, and muscle lactate recovery after intense exercise. It is also important to highlight that the application of WBV was considered safe, and no adverse effects were observed throughout the current study.

The existing literature on WBV in horses has primarily focused on the effects of WBV on clinical and blood parameters after a single session (43–45), neuromuscular activation (26), use as a warming-up exercise (26), its impact on lameness (24, 44), symmetry and muscle area (25, 46), hoof growth (47), stride length (44, 45), bone mineral content (45), postural stability (25), and thoracolumbar pain (25). No studies in horses have delved into the effects of an acute WBV session as a method to stimulate the transfer of lactate between white, glycolytic muscle fibers, which produce lactate, and red, oxidative muscle fibers, which consume lactate, within the working muscle (48) and a decrease in its concentration from the bloodstream after a high-intensity exercise session, which could help improve the body's acid-base balance (49).

The exercise bout was performed above the individual lactate threshold intensity, leading to increased muscular lactate production, inducing hyperlactatemia. One important performance-related marker is the lactate threshold, which correlates strongly with endurance performance and has proven sensitive to different acute exercise prescriptions. It characterizes the boundary between the heavy and severe exercise intensity domains, representing the threshold at which fast-twitch type II muscle fibers are more activated and the glycolytic pathway hyperactivate lactate production (50). This approach may provide a standard design for further studies focusing on the relationship between whole-body vibration and the acceleration of lactatemia and acid-base status recovery in exercising horses.

The use of WBV has increased in the equestrian industry to improve the performance and health of horses. However, little published research supports its use, and it is unclear what vibration magnitude is required for noticeable changes. The studies have shown mixed results, with some studies showing beneficial effects, others showing no effect and others showing adverse effects. The varying results are caused by differences in study protocols and designs and a lack of standardization in the physical aspects of the device, such as frequency, amplitude, and oscillation magnitude (18, 26, 51). In most studies involving horses, crucial details about vibratory intervention are often missing, such as the type and direction of vibration, the actual frequency, and the extent of vibration (displacement). This lack of information makes it difficult to compare data. However, these parameters are essential for evaluating vibratory intervention (51, 52).

During intense exercise, increased sympathetic activity in the nucleus tractus solitaries leads to elevated secretion of adrenal hormones (53). From an integrative perspective, the AIEB promoted transient adjustment in the homeostasis of the physiological variables studied herein, such as hyperlactatemia, mild acidosis, hyperglycemia, positive cardiac chronotropic, and elevated body temperature across the three recovery strategies trialed. Studies in humans have shown lower lactate concentrations in individuals who cooled down with WBV (2, 4, 5), indicating that WBV can lead to more efficient recovery from exercise attributable to continuous stimulation of blood vessels in the muscle. This stimulus could increase the use of lactate as energy for activated cardiac muscle and skeletal muscles and cause faster distribution of lactate to the liver (2). The findings indicated no difference between recovery strategies, demonstrating that the WBV protocol was ineffective in enhancing muscle and plasma lactate clearance.

Another way to accelerate lactate clearance would be via lactate transporters (MCTs). This mechanism would assist in a reduction of the plasma lactate concentration, which has been speculated to help the lactate outflow from type II muscle fibers (54). WBV also did not modify the MCT1 and MCT4 protein gluteus medius content, mainly expressed in oxidative and glycolytic fibers, respectively. It should be noted that this mechanism could help lactate exchanges between white-glycolytic and red-oxidative fibers and extracellular spaces (48, 55). Although muscle contractile activities can stimulate the gene and protein expression of MCTs in horses (56), a few studies have examined the effects of a single aerobic/anaerobic burst on MCTs (38). As traditionally reported, these authors found that both MCT1 and MCT4 increased following a single bout of maximal incremental exercise test. Aside from horses, this finding has already been described in rodents (57, 58) and humans (59). WBV did not alter lactate shutting through monocarboxylate transporters. Unexpectedly, MCT4 content decreased after AIEB in all recovery scenarios. Similarly, a human study reported that high-intensity exercise acutely decreased the amount of MCT4 protein in the sarcoplasm (60). These fortuitous findings may be caused by differences in exercise intensity, duration, testability, or sample preparation methods (61). Furthermore, it has been demonstrated that the expression of MCT is influenced by the breed of the horse (62, 63).

The results obtained showed the interference of exercise on blood gases and electrolytes, being that the AIEB protocol was of high intensity, characterized by the mobilization of the glycolytic pathway with the development of hyperlactatemia and metabolic acidosis, considering the findings of plasma lactate, pH, BE_ecf_, and SID. High-intensity exercise leads to sharp increases in [H^+^], which leads to decreased pH and reduced ${HCO}_{3}^{-}$ and tCO_2_ (64). Racehorses that underwent 2 min of high-intensity trotting showed decreased SID on jugular venous blood, mainly responsible for the observed plasma acidosis. The reduction in SID resulted from increased lactate, Na^+^, K^+^, and Cl^−^ (65, 66). The decrease in SID observed in the present study was mainly induced by the increase in lactate production caused by intense exercise.

Our study found no changes in PvCO_2_, consistent with a former study on intense treadmill exercise that suggested compensatory hyperventilation (67). In this study, ${HCO}_{3}^{-}$ values after exercise were lower than baseline values, like what Miranda et al. (67) observed, compatible with buffering mechanisms (68). The increase in Hct and Hb after AIEB is attributable to the release of the splenic reserve of red blood cells, mediated by the action of catecholamines in response to intense exercise (6), despite the changes observed after exercise. The results showed no differences in recovery strategies for blood gases and electrolytes, indicating that the recovery methods used did not affect the body's compensatory response to moderate metabolic acidosis caused by exercise.

Some studies have indicated that WBV can control glycaemia in humans (69, 70) and rodents (11, 71). The current study did not indicate that WBV could favor such an effect. The only thing that influenced plasma glucose was AIEB, which increased the bioavailability of this energy substrate for skeletal muscles. This finding can be explained by the increased activity of hormones that regulate energy metabolism, such as catecholamines and glucagon. These, when released, promote hepatic glycogenolysis and neoglycogenesis, an essential mechanism for maintaining plasma glucose concentrations during exercise (66).

After the recovery methods, the WBVG and SG showed lower HR compared to the TG, which can be explained by the fact that WBV is considered a form of passive recovery. However, there was no difference between the WBVG and SG, indicating that the VP turned off had the same effect as the equipment turned on. In adult men subjected to maximal exercise, a more pronounced reduction in HR was observed when recovering with an association of light exercise and WBV, compared to light exercise alone (4) and recovery with squats in VP on, compared to passive rest or only squats (5). These findings indicate that WBV led to a quicker heart rate recovery, a result not seen with the protocol used in the current study.

In the present study, it was possible to observe an increase in rectal temperature after AIEB. This variable increased even more after all interventions, around 15 min after the end of AIEB, in all groups. The increase in rectal temperature after exercise is significantly correlated with exercise (37). Corroborating these results, a study that evaluated different types of cooling with water after medium-intensity exercise observed an increase in rectal temperature, which remained elevated for up to 30 min after exercise, and the use of cooling with water as a form of recovery did not influence the decrease in post-exercise rectal temperature (31). Therefore, WBV did not accelerate the cooling down of this cohort of horses.

The use of mechanical vibration as an alternative to active exercise is of increasing interest. The effect of WBV on vital parameters, muscles and bones is investigated and it is widely suggested that WBV may be an alternative to resistance training for stimulation of the musculoskeletal system (43), which could make the technique an important training option, aiming to reduce the time of animals on the track, especially those with previous injuries and which would have a greater chance of recurrence. With the current search for alternatives to improve athletic capacity in the horse industry, it is not uncommon for participants to be mesmerized by the miraculous effects of therapies on the market. Companies that produce vibrating stimulation plates claim results such as increase/maintenance of muscle mass, reduction of injuries, faster healing, improved balance, and increased circulation, among others. The effectiveness of WBV as a training method, however, is quite controversial, and the results presented here did not indicate any effect on the physiological variables analyzed. In a study that investigated the effects of warming up with WBV in horses, the results showed that WBV can be seen as a passive movement of the limbs and trunk, as a type of relaxation of the locomotor system, without any active involvement (26).

More studies are needed in horses, especially regarding different vibration patterns, as the transmission of vibration through the equine body is poorly understood and is a fundamental consideration in interpreting the effects of WBV. A reduction in amplitude observed in the dorsal compared to the extremities of horses' bodies denotes that vibration transmission is greatly attenuated and may not be effective in provoking a physiological response, such as increased lactate removal from muscles, in the upper part of the body (72). To date, the use of WBV in horses is primarily based on results reported in human or rodent literature. However, horses are the largest animals on which WBV has been tested, and this size difference may impact vibration transmission. Furthermore, a possibly significant difference between WBV in men and horses is body posture during vibration training (24, 26). Moreover, most studies on WBV in humans involve performing other exercises on the VP (4, 5, 8), which, to date, is not possible to be carried out in the equine species.

While the study provides valuable insights, it is important to acknowledge that it has some limitations. For instance, there were a small number of horses involved, although this was enough for the statistical analysis. Additionally, using a single WBV session in the protocol may not have been enough to induce significant changes. It is possible that different results could be observed with longer periods of training using WBV. Also, the way vibrational transmission occurs throughout the horse's body is poorly understood; therefore, electromyography would be necessary to estimate how muscle activation occurs using WBV. Furthermore, although the gluteus medius muscle is the largest muscle in the horse and of great importance in its locomotion, showing great propulsive activity in the gaits during the exercise (73–75), it may be beneficial to assess other muscles in the distal thoracic or pelvic limbs, such as the triceps or semitendinosus, as the effects of vibration may diminish when moving dorsally (72). Hence, it is important to study different protocols for sport horse training to better evaluate the effects of WBV as a recovery method after intense exercise.

## 5 Conclusion

The research demonstrated that a single session of whole-body vibration (WBV) does not improve recovery from hyperlactatemia or acid-base balance in horses after intense exercise. This insight offers veterinarians, researchers and riding instructors the opportunity to develop better recovery protocols for horses. Future studies should focus on other protocols and the potential benefits of chronic WBV applications for enhancing recovery after exercise in horses.

## Data Availability

The raw data supporting the conclusions of this article will be made available by the authors, without undue reservation.
