# Supplementary material for: Acute whole-body vibration as a recovery strategy did not alter the content of gluteus medius monocarboxylate-transporters, lactatemia, and acidosis induced by intense exercise in horses
Source: Front Vet Sci. 2025 Mar 6;12:1538195. doi: 10.3389/fvets.2025.1538195 (PMC11925038; doi:10.3389/fvets.2025.1538195)
Supplement: Supplementary file 1 [file Table_1.docx]

Supplementary Material

# Supplementary Tables

**Supplementary Table 1.** Standardized exercise test (SET) protocol to determine the velocity corresponding to the lactate threshold (VLT) in horses (n=8).

| Steps | Time (min) | Speed (m/s) | Inclination (%) |
| --- | --- | --- | --- |
| Warming-up 1 | 3 | 1.5 | 0 |
| Warming-up 2 | 2 | 2.5 | 5 |
| Step 1 | 2 | 4 | 5 |
| Recovery period 1 | 2 | 1.5 | 5 |
| Step 2 | 2 | 5 | 5 |
| Recovery period 2 | 2 | 1.5 | 5 |
| Step 3 | 2 | 6 | 5 |
| Recovery period 3 | 2 | 1.5 | 5 |
| Step 4 | 2 | 7 | 5 |
| Recovery period 4 | 2 | 1.5 | 5 |
| Step 5 | 2 | 8 | 5 |
| Recovery period 5 | 2 | 1.5 | 5 |
| Step 6 | 2 | 9 | 5 |
| Cooling-down | 5 | Individual* | 0 |

*Speed varied from 1.2 to 1.6 m/s.

**Supplementary Table 2.** External load of acute intense exercise bout (AIEB) for each of the eight horses submitted to the SET.

| **Horses** | H1 | H2 | H3 | H4 | H5 | H6 | H7 | H8 |
| --- | --- | --- | --- | --- | --- | --- | --- | --- |
| **VLT_V_ (m/s)** | 4.9 | **6.8** | **5.2** | 7.0 | **5.4** | 7.0 | 6.8 | **5.9** |
| **VLT_BI_ (m/s)** | **5.0** | 6.6 | 4.8 | **7.1** | 4.8 | **7.1** | **7.0** | 5.1 |

Velocities corresponding to the lactate threshold were determined using the visual method (VLT_V_) and the bi-segmented method (VLT_BI_). The velocities utilized during the AIEB are highlighted in bold. H, horses.

**Supplementary Table 3.** Means ± standard deviation of heart rate (HR) and plasma lactate concentration in horses after intense exercise in each experimental block.

| **Variables** | **Blocks** | | |
| --- | --- | --- | --- |
|  | 1 | 2 | 3 |
| **Lactate (mM)** | 8.27±3.95 | 7.79±5.29 | 6.43±1.03 |
| **HR (bpm)** | 200±11 | 195±11 | 195±10 |

**Supplementary Table 4.** Means ± standard deviation of heart rate (bpm) of horses submitted to an acute intense exercise bout (AIEB) and a 10-minute recovery period on the treadmill (TG), on a whole-body vibration session (WBVG), or the vibrating platform off (SG).

| **Groups** | **Moments** | | | | |
| --- | --- | --- | --- | --- | --- |
|  | **Baseline** | **A** | **BR** | **AR** | **10min** |
| **TG** | 39±4^a^ | 197±11^b^ | 79±14^c^ | 84±11^Ac^ | 53±16^a^ |
| **WBVG** | 43±9^a^ | 194±12^b^ | 75±13^c^ | 59±9^Ba^ | 49±6^a^ |
| **SG** | 40±6^a^ | 198±10^b^ | 79±10^c^ | 65±12^Bd^ | 53±10^ad^ |

A, after AIEB. BR, before recovery. AR, after recovery. 10min, 10 min after the end of recovery. Different lowercase letters indicate significant differences in the intragroup comparison at *P*<0.05. Different capital letters indicate differences in the intergroup comparison at *P*<0.05.

**Supplementary Table 5**. Means ± standard deviation of rectal temperature (ºC) of horses submitted to an acute intense exercise bout (AIEB) and a 10-minute recovery period on the treadmill (TG), on a whole-body vibration session (WBVG), or the vibrating platform off (SG).

| **Groups** | **Moments** | | |
| --- | --- | --- | --- |
|  | **Baseline** | **A** | **AR** |
| **TG** | 37.0±0.44^a^ | 37.8±0.59^b^ | 38.1±0.52^b^ |
| **WBVG** | 37.0±0.36^a^ | 37.8±0.29^b^ | 38.4±0.28^c^ |
| **SG** | 36.9±0.62^a^ | 38.1±0.47^b^ | 38.5±0.22^b^ |

A, after AIEB. AR, after recovery. 10min, 10 min after the end of recovery. Different lowercase letters indicate significant differences in the intragroup comparison at *P*<0.05.

**Supplementary Table 6**. Means ± standard deviation of plasma lactate and glucose of horses submitted to an acute intense exercise bout (AIEB) and a 10-minute recovery period on the treadmill (TG), on a whole-body vibration session (WBVG), or the vibrating platform off (SG).

| **Variables** | **Groups** | **Time points** | | | | | |
| --- | --- | --- | --- | --- | --- | --- | --- |
|  |  | **Baseline** | **A** | **BR** | **AR** | **10min** | **1h** |
| **Lactate**  **(mM)** | **TG** | 0.49±0,05^ac^ | 7.70±4.11^b^ | 6.41±4.58^a^ | 3.18±3.24^c^ | 2.20±2.00^c^ | 0.65±0.18^ac^ |
|  | **WBVG** | 0.49±0.03^a^ | 7.71±3.83^b^ | 6.24±3.51^c^ | 3.82±2.82^a^ | 2.64±1.97^a^ | 0.64±0.17^abc^ |
|  | **SG** | 0.52±0.09^ab^ | 7.92±5.22^a^ | 6.92±5.85^a^ | 4.65±5.31^b^ | 3.46±4.35^b^ | 0.77±0.58^ab^ |
|  |  |  |  |  |  |  |  |
| **Glucose**  **(mM)** | **TG** | 6.16±0.92^ab^ | 6.78±1.06^ab^ | 7.11±1.86^ab^ | 7.24±1.39^a^ | 6.99±1.33^ab^ | 6.50±1.15^b^ |
|  | **WBVG** | 6.03±0.56^ab^ | 6.61±0.81^ab^ | 7.62±1.53^a^ | 7.43±1.51^a^ | 7.41±1.46^a^ | 6.50±1.00^b^ |
|  | **SG** | 6.17±0.78^ab^ | 6.55±1.20^ab^ | 7.98±2.07^a^ | 7.63±1.74^a^ | 7.57±1.71^a^ | 6.44±1.57^b^ |

A, after AIEB. BR, before recovery. AR, after recovery. 10min, 10 min after the end of recovery. 1h, 1 hour after the end of recovery. Different lowercase letters indicate differences in the intragroup comparison at *P*<0.05.

**Supplementary Table 7**. Means ± standard deviation of MCT1 and MCT4 proteins content, in arbitrary units, in the gluteus medius muscle of horses submitted to an acute intense exercise bout (AIEB) and a 10-minute recovery period on the treadmill (TG), on a whole-body vibration session (WBVG), or the vibrating platform off (SG).

|  | **Groups** | **Moments** | | | |
| --- | --- | --- | --- | --- | --- |
|  |  | **Baseline** | **AR** | **3h** | **6h** |
| **MCT1** | **TG** | 0.067±0.034 | 0.044±0.022 | 0.038±0.023 | 0.056±0.011 |
|  | **WBVG** | 0.070±0.036 | 0.049±0.026 | 0.057±0.028 | 0.057±0.047 |
|  | **SG** | 0.055±0.042 | 0.047±0.023 | 0.055±0.035 | 0.067±0.046 |
|  |  |  |  |  |  |
| **MCT4** | **TG** | 0.443±0.259^a^ | 0.424±0.294^ab^ | 0.239±0.136^c^ | 0.267±0.162^bc^ |
|  | **WBVG** | 0.456±0.265^a^ | 0.512±0.381^ab^ | 0.317±0.173^c^ | 0.376±0.193^bc^ |
|  | **SG** | 0.468±0.368^a^ | 0.328±0.121^ab^ | 0.271±0.156^c^ | 0.264±0.168^bc^ |

AR, after recovery. 3h, 3 hours after recovery; 6h, 6 hours after recovery. Different lowercase letters indicate differences in the intragroup comparison at *P*<0.05.

**Supplementary Table 8**. Means ± standard deviation of the area under the curve of lactate, MCT1 and MCT4 proteins content, of horses submitted to an acute intense exercise bout (AIEB) and a 10-minute recovery period on the treadmill (TG), on a whole-body vibration session (WBVG), or the vibrating platform off (SG).

|  | **Groups** | **Mean ± SD** | **Median (range)** | **Confidence interval** |
| --- | --- | --- | --- | --- |
| **Lactate (mM)** | **TG** | 225 ± 146 | 177 (101-475) | 122 |
|  | **WBVG** | 226 ± 133 | 188 (108-517) | 111 |
|  | **SG** | 278 ± 278 | 188 (81.6-937) | 232 |
|  |  |  |  |  |
| **MCT1 (AU)** | **TG** | 2.3 ± 2.8 | 1.31 (0.022-8.32) | 2.59 |
|  | **WBVG** | 1.28 ± 1.08 | 1.19 (0.236-3.3) | 0.997 |
|  | **SG** | 1.16 ± 0.789 | 1.08 (0.178-2.43) | 0.73 |
|  |  |  |  |  |
| **MCT4 (AU)** | **TG** | 13.1 ± 9.12 | 11.5 (3.5-27.6) | 8.43 |
|  | **WBVG** | 14.9 ± 11.4 | 10.0 (3.28-32.1) | 10.5 |
|  | **SG** | 9.60 ± 4.54 | 8.65 (2.24-15.8) | 4.20 |

TG, recovery on the treadmill. WBVG, recovery on whole-body vibration session. SG, sham group, recovery on platform off.
